# Structural Stability, Mechanical, and Electronic Properties of Al_5_TM (TM = Mo, Nb, Os, Re, Ru, Ta, Tc, Ti) Intermetallics

**DOI:** 10.3390/nano15161221

**Published:** 2025-08-10

**Authors:** Jiaxiang Yang, Qun Wei, Jing Luo, Meiguang Zhang, Bing Wei

**Affiliations:** 1 School of Physics, Xidian University, Xi’an 710071, China; 2 College of Physics and Optoelectronic Technology, Baoji University of Arts and Sciences, Baoji 721016, China

**Keywords:** first-principles calculations, structural stability, electronic structures, mechanical properties

## Abstract

Al-based intermetallic compounds possess excellent mechanical and thermal properties, making them promising candidates for high-temperature structural applications. In this study, the structural stability, mechanical properties, and electronic characteristics of Al_5_TM (TM = Mo, Nb, Os, Re, Ru, Ta, Tc, Ti) intermetallic compounds were systematically investigated using first-principles calculations based on density functional theory. All alloys exhibit negative formation energy, indicating favorable thermodynamic stability. Elastic constant analysis shows that all compounds satisfy the Born stability criteria, confirming their mechanical stability. Among them, Al_5_Mo (205.9 GPa), Al_5_Nb (201.1 GPa), and Al_5_Ta (204.1 GPa) exhibit relatively high Young’s moduli, while Al_5_Os, Al_5_Re, and Al_5_Ru demonstrate large bulk moduli and good ductility. The high Debye temperatures of Al_5_Mo (600.5 K) and Al_5_Nb (606.7 K) suggest excellent thermal stability at elevated temperatures. Electronic structure analysis reveals that all alloys exhibit metallic behavior with no band gap near the Fermi level. The hybridization between TM-*d* and Al-3*p* orbitals enhances the covalent bonding between Al and TM atoms. This study provides theoretical guidance for the design and application of high-performance Al-based intermetallic compounds.

## 1. Introduction

High-temperature alloy materials exhibit excellent mechanical properties, oxidation resistance, and structural stability under extreme high-temperature conditions [1,2,3,4,5,6]. With the increasing demands for higher service temperatures and comprehensive performance in fields such as aerospace, gas turbines, and high-temperature structural components, the development of a new generation of lightweight high-temperature alloys has become a key research focus in materials science [7,8,9,10].

Compared with traditional Ni-based and Co-based high-temperature alloys, Al-based high-temperature alloys offer advantages such as low density, high strength, and superior corrosion resistance, making them a highly promising alternative material [11,12]. In recent years, the structural design and performance analysis of aluminum-based alloys have attracted widespread attention [13]. Wang et al. [14] predicted three new structures of IrAl_3_ and revealed the differences in mechanical and thermodynamic properties among these structures. Duan et al. [15] conducted a systematic investigation of Zr–Al binary intermetallic compounds and identified ZrAl_2_ as the most stable compound, while ZrAl exhibited the strongest anisotropy. Huang et al. [16,17,18] conducted a comprehensive study on the structural characteristics and mechanical properties of FeCrAl alloys. With ongoing research advancements, novel aluminum alloys have gradually become a focal point in high-temperature alloy studies [19,20,21,22]. Among various aluminum-based high-temperature alloys, Al-rich alloys have attracted widespread attention because the presence of a substantial amount of Al_2_O_3_ oxide films can significantly enhance high-temperature oxidation resistance. In the Al-rich system, Liang et al. [23] successfully fabricated Al_4_W/Al_12_W composites in situ via an infiltration method under vacuum conditions. Wang et al. [24] investigated the mechanical and thermodynamic properties of Al_4_W, Al_5_W, and Al_12_W under pressure. Pan [25] studied the structural stability, elastic properties, and thermodynamic behavior of hexagonal and cubic Al_5_Mo alloys. The results showed that hexagonal Al_5_Mo exhibits good thermodynamic and kinetic stability, with mechanical properties superior to those of cubic Al_5_Mo. Wang and Chen [26] studied the mechanical properties, electronic properties, and Debye temperature of Al_5_Mo under pressure. In addition, Pan and Wei [27,28] predicted and studied the structural stability, elastic anisotropy, and melting point of the Al_5_W alloy. Luo et al. [29] predicted five structures of Ru_2_Al_5_ and analyzed their mechanical and electronic properties, confirming that the experimentally synthesized Ru_2_Al_5_ phase adopts an orthorhombic *Pmmn* crystal structure.

The Al_5_TM alloy system is an emerging class of aluminum-based high-temperature alloys. In this system, transition metal elements form stable intermetallic compounds with aluminum, exhibiting excellent thermal stability and high melting points. These alloys maintain good structural integrity and mechanical properties even under high-temperature conditions. Although previous studies have provided a solid theoretical foundation for aluminum-based alloys [30,31,32], theoretical research on the Al_5_TM alloy system remains relatively limited. In this work, we systematically investigated the stability, mechanical properties, Debye temperature, and electronic structure of Al_5_TM (TM = Mo, Nb, Os, Re, Ru, Ta, Tc, Ti) intermetallic compounds using first-principles calculations. The results indicate that all eight Al_5_TM alloys exhibit excellent stability and metallic conductivity. Al_5_Mo exhibits the best thermodynamic stability. Among them, the Al_5_Mo (205.9 GPa), Al_5_Nb (201.1 GPa), and Al_5_Ta (204.1 GPa) alloys exhibit relatively high Young’s moduli. Al_5_Mo (600.5 K) and Al_5_Nb (606.7 K) show relatively high Debye temperatures. These findings provide insights into the potential applications of Al_5_TM compounds in high-performance materials, offering a theoretical foundation for the design and development of a new generation of lightweight high-temperature alloys.

## 2. Structural and Computational Information

In this study, the stable *R*32-Al_5_W structure [28] was used as the prototype, with lattice parameters *a* = 4.9393 Å, *c* = 13.1864 Å. Eight Al_5_TM (TM = Mo, Nb, Os, Re, Ru, Ta, Tc, Ti) alloy structures were constructed by substituting the W atom with transition metal atoms for further investigation. First-principles calculation methods are commonly used to evaluate the structural stability and mechanical properties [33,34,35,36,37,38], enabling accurate predictions of material stability, elastic properties, and electronic behavior based on their electronic structure. We performed calculations of the phonon dispersion, elastic moduli, and band structures for all Al_5_TM structures using the Vienna Ab initio Simulation Package (VASP) [39]. The electronic exchange-correlation energy was described using the generalized gradient approximation (GGA) with the Perdew–Burke–Ernzerhof (PBE) functional [40], as implemented in the VASP code. In addition, to ensure the accuracy of the computational results, the plane-wave cutoff energy for all Al_5_TM structures was set to 600 eV, achieving good total energy convergence with a convergence criterion of 1 × 10^−5^ eV/atom. The *k*-point sampling grid for the Brillouin zone was set to 15 × 15 × 4. The lattice parameters and atomic positions were fully relaxed after structural optimization. The phonon dispersion relations of the Al_5_TM structures were obtained through calculations using the PHONOPY package [41]. The formation energy of the Al_5_TM alloy can be calculated using the following formula [28]:

(1) $$E_{f}\;=\;\left\{E\left({Al}_{5}TM\right)\;-\;E\left(TM\right)\;-\;5E(Al)\right\}/6$$

where *E*(Al_5_TM) represents the total energy of the Al_5_TM. *E*(Al) and *E*(TM) represent the total energy of metallic aluminum and the transition metal (TM), respectively.

## 3. Results and Discussion

**Figure 1:**
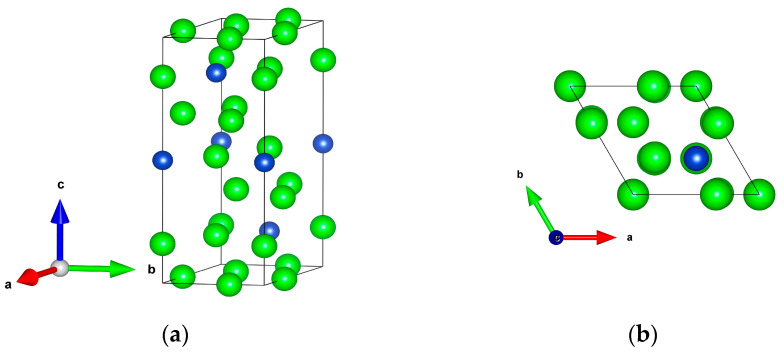
Crystal structure of Al_5_TM (TM = Mo, Nb, Os, Re, Ru, Ta, Tc, Ti): (**a**) three-dimensional view; (**b**) top view. The blue and green balls are TM and Al atoms, respectively.

The crystal structure of Al_5_TM belongs to the trigonal crystal system and is in the *R*32 phase, with its structure shown in Figure 1. Based on the structural diagrams, it is evident that the transition metal (TM) atoms occupy highly symmetric positions within the unit cell and are coordinated by Al atoms in the form of polyhedral structures. In the trigonal unit cell, atoms are stacked in layers along the c-axis, following the close-packed arrangement typically observed in intermetallic compounds. This layered structure, coupled with the dense distribution of Al atoms, contributes to a compact and stable overall configuration. The TM atoms situated between the layers provide additional structural support along the vertical (*c*-axis) direction.

The formation energy of the Al_5_TM structure was calculated using Equation (1). A negative formation energy indicates that the structure is thermodynamically stable, whereas a positive value suggests instability. Table 1 presents the calculated lattice parameters and formation energy of the Al_5_TM alloys. The calculation results indicate that the formation energies of all Al_5_TM alloys are negative, indicating that these structures possess thermodynamic stability. And the order of formation energy is Al_5_Re > Al_5_Ta > Al_5_Os > Al_5_Ti > Al_5_Nb > Al_5_Ru > Al_5_Tc > Al_5_Mo, indicating that among the Al_5_TM alloys obtained through atomic substitution, Al_5_Mo exhibits the highest thermodynamic stability. It is worth noting that the formation energy of the *R*32 phase of Al_5_Mo is lower than that of the *P*6_3_ phase [26], indicating that the *R*32 phase of Al_5_Mo possesses better thermodynamic stability.

Due to their potential applications in high-temperature environments, Al_5_TM alloys have received widespread attention for their mechanical properties and structural integrity. To evaluate the mechanical performance of Al_5_TM, particular emphasis must be placed on their elastic properties. For trigonal Al_5_TM alloys, mechanical stability is conventionally determined by the elastic stiffness constants *C_ij_*, which must satisfy the Born stability criteria to ensure resistance to mechanical deformation under external stress [42]. For trigonal crystal systems, the criteria for mechanical stability are:

(2) $$C_{44}\;>\;0$$

(3) $$C_{11}-{|C}_{12}|>0$$

(4) $$C_{33}\left(C_{11}\;+\;C_{12}\right)-2C_{13}^{2}>0$$

(5) $$C_{44}\left(C_{11}-C_{12}\right)-2C_{14}^{2\;}>0$$

For Al_5_TM alloys, accurately obtaining their elastic constants through first-principles calculations provides a critical basis for revealing their anisotropic mechanical properties. As shown in Table 2, the calculated elastic constants of the Al_5_TM alloys satisfy the relevant mechanical stability criteria, indicating that these structures are mechanically stable. For the Al_5_Os and Al_5_Ru alloys, the *C*_11_ values exceed the corresponding *C*_33_ values, indicating higher linear incompressibility along the [100] crystallographic direction and enhanced resistance to axial compression relative to the [001] direction. This anisotropic behavior is particularly pronounced in Al_5_Os, which exhibits the most significant *C*_11_ > *C*_33_ disparity, suggesting superior compressive strength along the *a*-axis. Such directional dependence of compressive resistance is likely attributed to stronger interatomic bonding aligned predominantly along the [100] direction, whereas weaker bonds are more likely oriented perpendicular to the [001] plane. Furthermore, for the Al_5_Mo, Al_5_Nb, Al_5_Ta, Al_5_Tc, and Al_5_Ti alloys, the elastic constant *C*_33_ exceeds *C*_11_, indicating greater linear incompressibility along the [001] crystallographic direction and enhanced resistance to uniaxial compression compared to the [100] direction. Notably, Al_5_Re exhibits *C*_11_ = *C*_22_ = *C*_33_, implying isotropic compressive behavior along all three principal crystallographic axes. The elastic constants *C*_44_ and *C*_66_ represent the shear modulus within the elastic stiffness matrix and reflect the material’s resistance to shear deformation. They play a critical role in characterizing the elastic anisotropy of crystalline materials. The relatively low *C*_44_ values of Al_5_Os and Al_5_Ru, along with the low *C*_66_ value of Al_5_Ti, indicate limited resistance to shear deformation, suggesting that these materials are more prone to distortion under applied shear stress. In contrast, Al_5_Mo, Al_5_Nb, and Al_5_Ta exhibit comparatively higher *C*_44_ and *C*_66_ values, reflecting higher shear modulus and thus stronger resistance to shear deformation.

**Figure 2:**
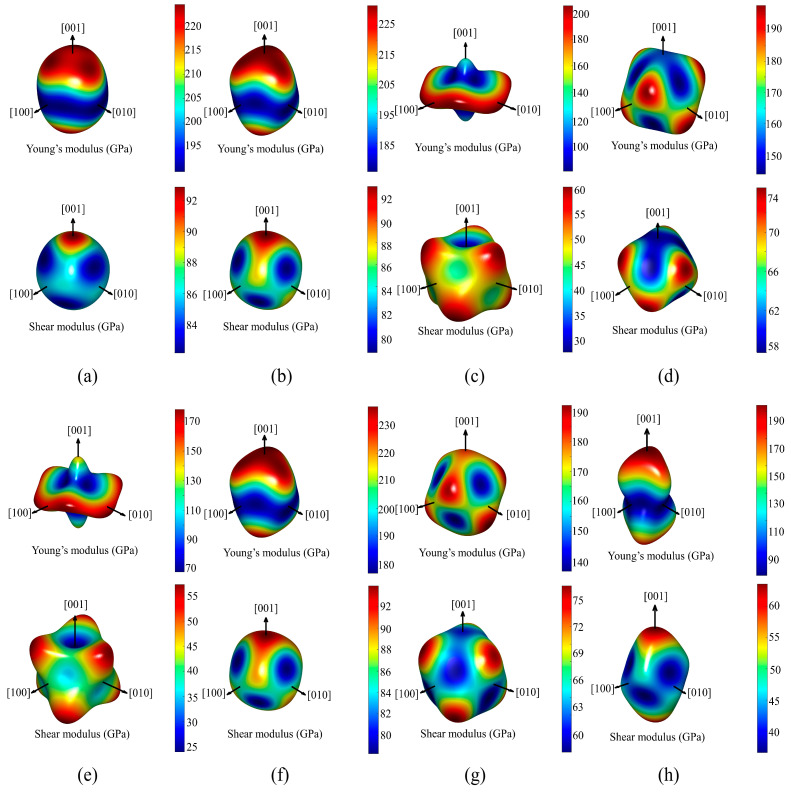
Three-dimensional anisotropy diagrams of Young’s modulus and shear modulus for the Al_5_TM alloys: (**a**) Al_5_Mo, (**b**) Al_5_Nb, (**c**) Al_5_Os, (**d**) Al_5_Re, (**e**) Al_5_Ru, (**f**) Al_5_Ta, (**g**) Al_5_Tc, and (**h**) Al_5_Ti.

In order to thoroughly assess the mechanical properties of Al_5_TM alloys, we calculated the elastic modulus, Poisson’s ratio (*v*), and the *B*/*G* ratio. The Young’s modulus, elastic modulus, and shear modulus were calculated using the Voigt–Reuss–Hill averaging scheme [43]. The calculation results are listed in Table 3. The Young’s modulus of the Al_5_TM alloys varies significantly, ranging from 117.9 to 205.9 GPa, underscoring the substantial impact of different transition metal elements on the elastic stiffness. Notably, the Al_5_Mo, Al_5_Nb, and Al_5_Ta alloys exhibit the highest Young’s modulus values of 205.9 GPa, 201.1 GPa, and 204.1 GPa, respectively, indicating superior resistance to deformation. This high stiffness is primarily attributed to the ability of Nb, Ta, and Mo atoms to participate in covalent bond formation through their *d*-orbital electrons, which significantly enhances the bonding interactions between TM–Al and TM–TM. In contrast, alloys such as Al_5_Ru, Al_5_Ti, and Al_5_Os exhibit lower Young’s modulus values of 117.9 GPa, 121.7 GPa, and 133.3 GPa, respectively, indicating weaker interatomic bonding within their crystal structures. This difference may originate from the characteristics of the *d*-electron configurations of the TM elements. For example, the d orbitals of Ru and Os are nearly fully occupied, which weakens the directionality of covalent bonds and thereby affects the elastic response of the material under applied stress. Furthermore, Al_5_Re and Al_5_Tc exhibit intermediate Young’s modulus values of 169.8 GPa and 165.2 GPa, respectively. Overall, the elastic properties of Al_5_TM alloys are influenced not only by the atomic volume and electronic structure of the TM elements but also by their bonding interactions with Al atoms within the crystal lattice. Figure 2 shows the three-dimensional anisotropy diagrams of Young’s moduli and shear moduli for the Al_5_TM alloys.

The bulk modulus *B* and shear modulus *G* are important parameters for analyzing the mechanical properties of materials [44]. The bulk modulus *B* and shear modulus *G* reflect a material’s resistance to volumetric compression and shear deformation, respectively. Together, these two elastic moduli govern the overall stiffness and structural stability of the material [45]. As shown in the data presented in Table 3, Al_5_Os, Al_5_Re, and Al_5_Ru exhibit relatively high bulk moduli of 130.3 GPa, 134.6 GPa, and 118.3 GPa, respectively, indicative of strong resistance to volumetric compression. Nevertheless, their shear moduli are relatively low, being 50.1 GPa, 65.8 GPa and 44.2 GPa, respectively, indicating that their resistance to shear deformation is limited. This indicates that although atomic bonding is relatively dense, the materials demonstrate limited resistance to shear deformation, highlighting pronounced elastic anisotropy. In contrast, Al_5_Mo, Al_5_Nb, and Al_5_Ta display both high bulk moduli (116.3 GPa, 106.3 GPa, and 109.9 GPa, respectively) and high shear moduli (85.5 GPa, 84.9 GPa, and 85.7 GPa, respectively), indicating their excellent mechanical performance in resisting both volumetric compression and shear deformation.

Furthermore, the *B*/*G* ratio is employed as an evaluation criterion to assess the brittleness and ductility of Al_5_TM alloys. According to Pugh’s criterion, materials with a *B*/*G* ratio below 1.75 exhibit brittle behavior, whereas those with a ratio above 1.75 are considered ductile [46]. The analysis results indicate that Al_5_Os (2.60), Al_5_Re (2.04), and Al_5_Ru (2.68) exhibit pronounced ductile behavior, whereas Al_5_Nb (1.25), Al_5_Mo (1.36), and Al_5_Ta (1.28) display characteristic brittle behavior. In summary, Al_5_TM alloys maintain high compressive performance while their shear rigidity and ductility are significantly influenced by the TM elements. Poisson’s ratio describes the relationship between transverse strain and axial strain during the deformation of a material under applied stress [47]. For crystal structures, Poisson’s ratio characterizes the deformation response along different crystallographic directions, demonstrating its elastic anisotropy. Poisson’s ratio reflects the bonding characteristics between atoms in the crystal structure, where the bonding strength and directionality directly determine the material’s ability to resist deformation under external forces. According to linear elasticity theory, the mechanical stability criterion for materials requires that Poisson’s ratio lies between −1 and 0.5 [48]. The Poisson’s ratio results presented in Table 3 indicate that the investigated Al_5_TM alloys exhibit good mechanical stability under elastic deformation.

The thermal stability of materials at elevated temperatures is a critical criterion for assessing their high-temperature performance [49,50,51]. In this study, the Debye temperature is introduced to further evaluate the thermodynamic properties of Al_5_TM alloys. This parameter reflects the characteristics of phonon vibrations in solids and to a certain extent reveals the dynamic properties of the material. The calculation formula is as follows [52]:

(6) $$\theta_{D}\;=\;{\frac{h}{k_{B}}\left[\frac{3n}{4\pi }\left(\frac{N_{A}\rho }{M}\right)\right]}^{\frac{1}{3}}\;{\times \;v}_{m}$$

where $k_{B}$, h, and $N_{A}$ represent the Boltzmann constant, Planck’s constant, and Avogadro’s constant, respectively. *n* represents the number of atoms, and *M* represents the molar mass. *v_m_* represents the average sound velocity, which is calculated using the following equation:

(7) $$v_{m}\;=\;{\left[\frac{1}{3}\left(\frac{2}{v_{t}^{3}}\;+\;\frac{1}{v_{l}^{3}}\right)\right]}^{-\frac{1}{3}}$$

where $v_{l}$ and $v_{t}$ represent the longitudinal and transverse sound velocities, respectively, and are calculated using the following equations [53]:

(8) $$v_{l}\;=\;{\left(\frac{3B\;+\;4G}{3\rho }\right)}^{\frac{1}{2}}$$

(9) $$v_{t}={\left(\frac{G}{\rho }\right)}^{\frac{1}{2}}$$

The velocity of sound in a material is typically related to its elastic modulus and density. A higher sound velocity indicates stronger interatomic interactions, reflecting greater stiffness and superior elastic properties. The sound velocity data presented in Table 4 reveal the significant influence of different transition metal elements on the longitudinal velocity, transverse velocity, and average sound velocity of the Al_5_TM alloys. The Al_5_Mo and Al_5_Nb alloys exhibit high average sound velocities of 5070 m/s and 5174 m/s, respectively, indicating their excellent elastic and stiffness properties. Additionally, Al_5_Ti, Al_5_Ta, and Al_5_Tc also show relatively high average sound velocities (4388–4405 m/s), reflecting a well-balanced mechanical performance. These differences in sound velocity mainly originate from the varying effects of different transition metal elements on the stiffness of the crystal structure and the strength of interatomic bonding.

**Figure 3:**
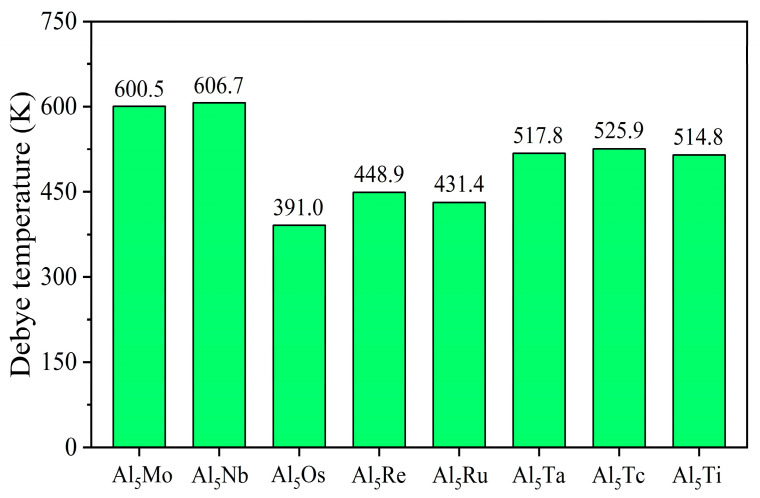
Debye temperature of Al_5_TM alloys.

Figure 3 shows the calculated Debye temperatures of Al_5_TM alloys, revealing significant differences in Debye temperatures among various aluminum-based alloys. As shown in the figure, the Al_5_Mo and Al_5_Nb alloys exhibit relatively high Debye temperatures (600.5 K and 606.7 K), indicating that these two alloys possess superior thermodynamic properties. It is worth noting that all Al_5_TM alloys exhibit favorable thermodynamic properties. Their excellent performance stems from the interatomic interactions and vibrational frequencies within the alloys.

**Figure 4:**
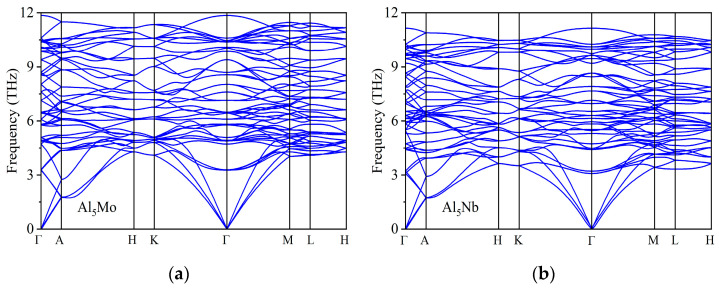

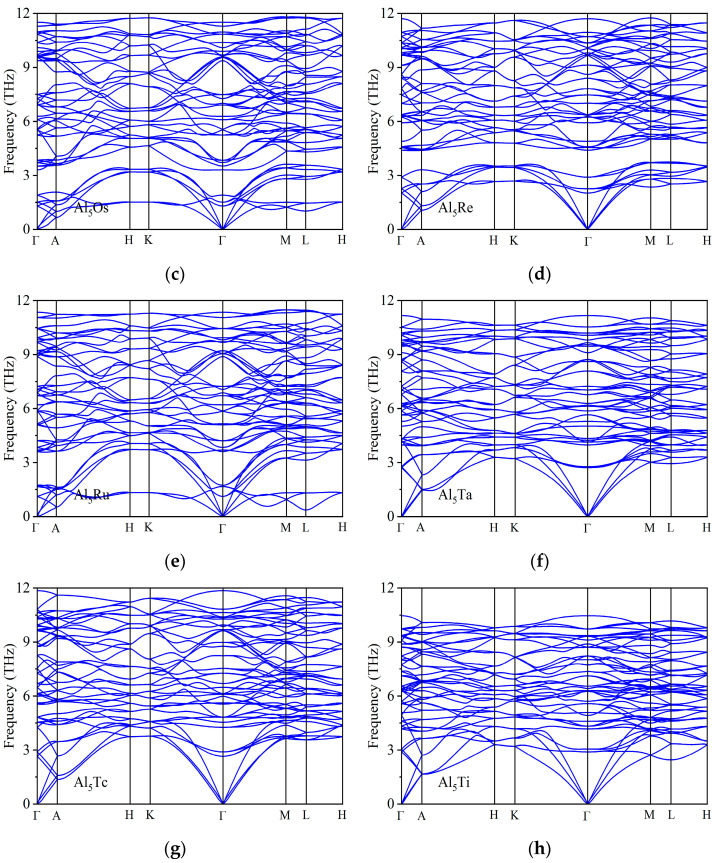
Calculated phonon dispersion curves of Al_5_TM alloys: (**a**) Al_5_Mo, (**b**) Al_5_Nb, (**c**) Al_5_Os, (**d**) Al_5_Re, (**e**) Al_5_Ru, (**f**) Al_5_Ta, (**g**) Al_5_Tc, and (**h**) Al_5_Ti.

Dynamical stability is a fundamental criterion for evaluating the overall structural stability of crystalline materials [54]. To this end, we investigated the dynamical stability of the Al_5_TM structures to comprehensively assess their structural stability. Phonon dispersion analysis, which provides insight into atomic vibrational modes and energy distributions within the crystal lattice, was employed to examine their dynamical behavior [55]. The dynamical stability of Al_5_TM alloys was systematically assessed through a detailed analysis of their phonon dispersion relations and atomic vibrational frequencies. Figure 4 presents the computed phonon spectra for these alloys. For dynamically stable structures, the phonon spectrum contains no imaginary frequencies. It is worth emphasizing that no imaginary frequencies are present in the phonon spectra of all Al_5_TM alloys, indicating that these alloys exhibit excellent dynamical stability.

**Figure 5:**
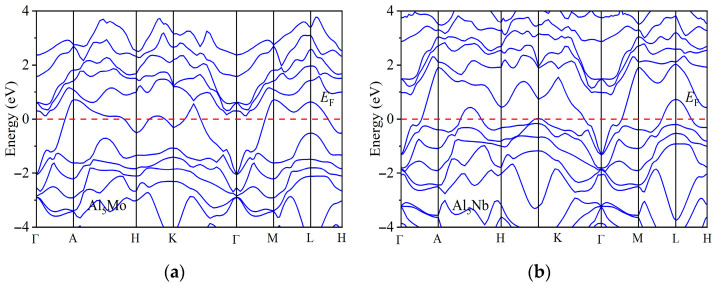

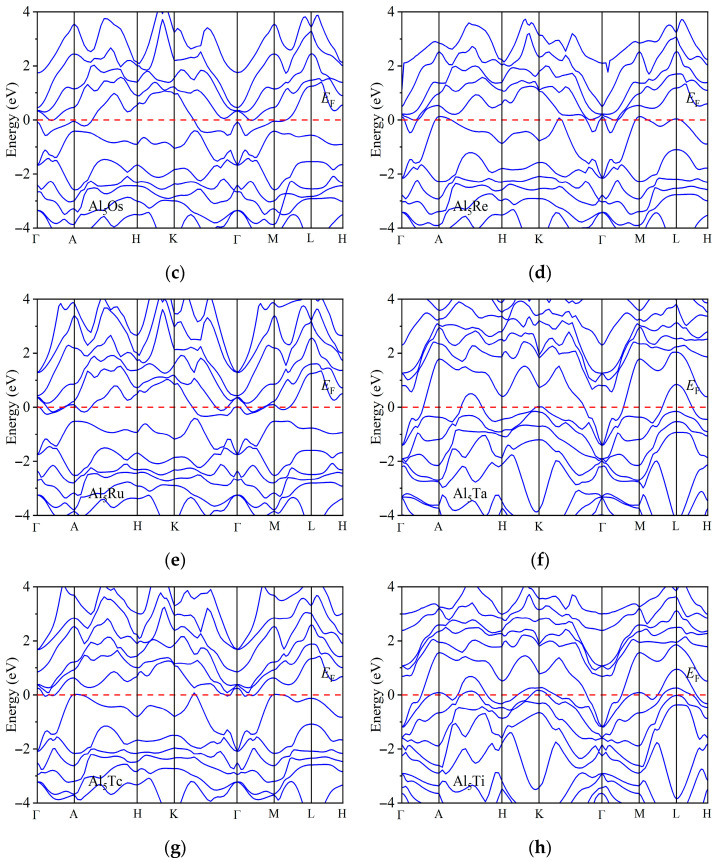
Calculated band structures of Al_5_TM alloys: (**a**) Al_5_Mo, (**b**) Al_5_Nb, (**c**) Al_5_Os, (**d**) Al_5_Re, (**e**) Al_5_Ru, (**f**) Al_5_Ta, (**g**) Al_5_Tc, and (**h**) Al_5_Ti.

A systematic analysis of the band structures of the Al_5_TM alloys was carried out to gain a deeper understanding of their electronic properties. Figure 5 presents the calculated band structures of the Al_5_TM alloys along the high-symmetry path Γ → A → H → K → Γ → M → L → H. The specific coordinates of each high-symmetry point are Γ (0.0 0.0 0.0), A (0.0 0.0 0.5), H (−0.333 0.667 0.5), K (−0.333 0.667 0.0), M (0.0 0.5 0.0), L (0.0 0.5 0.5). The band structure reveals that multiple bands cross the Fermi level, and no evident band gap is observed, indicating that the Al_5_TM alloys exhibit typical metallic behavior. Based on the distribution of the electronic band structure, it can be inferred that the d electrons of the transition metal elements make the most significant contribution near the Fermi level. Further analysis of the distribution characteristics of electronic density of states confirmed this inference result.

**Figure 6:**
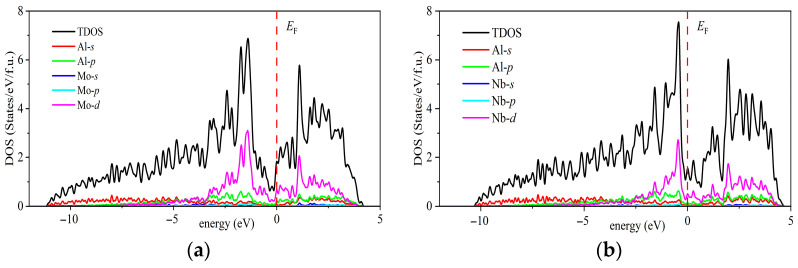

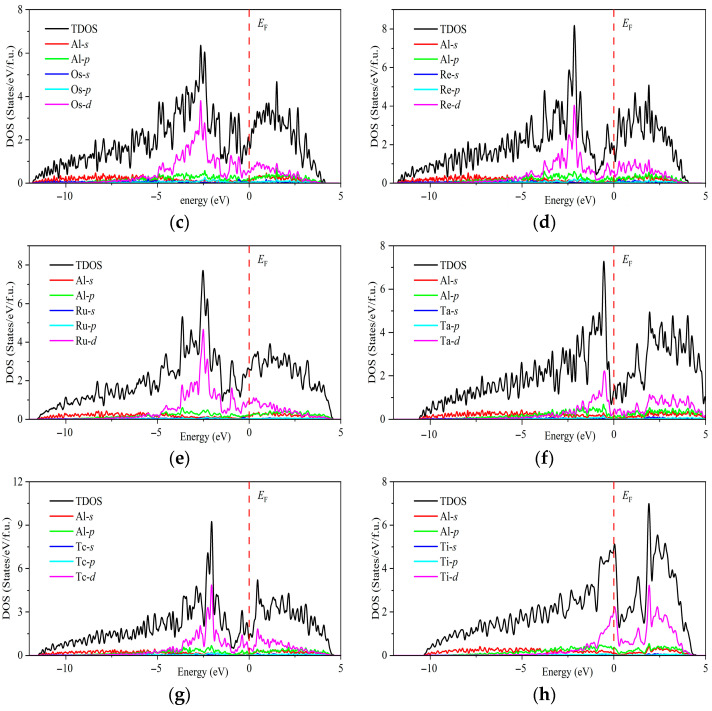
Projected and total density of states of Al_5_TM alloys: (**a**) Al_5_Mo, (**b**) Al_5_Nb, (**c**) Al_5_Os, (**d**) Al_5_Re, (**e**) Al_5_Ru, (**f**) Al_5_Ta, (**g**) Al_5_Tc, and (**h**) Al_5_Ti.

The density of states (DOS) refers to the number of electronic states available per unit energy interval that can be occupied by electrons, reflecting the distribution of electrons at specific energy levels. Projected density of states (PDOS) is a further analysis of the total DOS, revealing the contributions of specific elements or atomic orbitals within a certain energy range. The total density of states (TDOS) and PDOS of the Al_5_TM alloys are shown in Figure 6, providing insight into their electronic structure and bonding characteristics. It is evident from the figure that the TDOS at the Fermi level (*E*_F_) is nonzero for all alloys, indicating that these compounds exhibit typical metallic behavior. In terms of electronic state distribution, the *d* orbitals of the transition metal elements dominate across the entire energy range, particularly near the Fermi level. The *d* orbitals of the transition metals make the most significant contribution to the TDOS and are the key factor determining the electronic properties of the alloys. In contrast, the contributions from the Al *p* and *s* orbitals near the Fermi level are relatively minor, indicating that Al atoms primarily serve a structural role in the alloy and participate in bonding indirectly. The *p* and *s* orbitals of Al exhibit a certain degree of hybridization with the *d* orbitals of the TM elements in the lower energy region of the band structure. In particular, the hybridization between the TM-*d* and Al-*p* orbitals is more pronounced, enhancing the covalent bonding interactions between Al and TM atoms. In summary, the density of states analysis not only confirms the metallic nature of the Al_5_TM alloys but also highlights the crucial role of TM *d* orbitals in determining their electronic structure and bonding behavior. The hybridization between aluminum atoms and transition metal elements enhances the electronic interactions and bonding strength within the material, significantly improving the overall performance of the Al_5_TM alloys.

## 4. Conclusions

In this study, first-principles calculations based on density functional theory were employed to systematically investigate the structural stability, mechanical properties, and electronic characteristics of Al_5_TM (TM = Mo, Nb, Os, Re, Ru, Ta, Tc, Ti) intermetallic compounds. All Al_5_TM alloys exhibit negative formation energies, indicating good thermodynamic stability. Among them, Al_5_Mo has the lowest formation energy, making it the most stable structure within the system. Compared with the *P*6_3_ phase, the *R*32 phase of Al_5_Mo possesses a lower formation energy, demonstrating superior thermodynamic stability. The calculated elastic constants and moduli indicate that all Al_5_TM alloy structures satisfy the Born mechanical stability criteria, exhibiting good mechanical stability. Among them, Al_5_Mo, Al_5_Nb, and Al_5_Ta exhibit high Young’s and shear moduli, indicating excellent rigidity and resistance to shear deformation. Al_5_Os, Al_5_Re, and Al_5_Ru exhibit high bulk moduli, indicating excellent resistance to volumetric compression. According to Pugh’s criterion, Al_5_Os, Al_5_Re, and Al_5_Ru exhibit good ductility, whereas Al_5_Nb, Al_5_Mo, and Al_5_Ta show pronounced brittle characteristics. Phonon dispersion analysis reveals no imaginary frequencies, indicating that all Al_5_TM alloys possess good dynamical stability. The Debye temperatures of Al_5_Mo and Al_5_Nb reach 600.5 K and 606.7 K, respectively, indicating excellent thermal stability at high temperatures. The electronic states near the Fermi level primarily originate from the *d* orbitals of the transition metals and exhibit strong hybridization with the Al-3*p* orbitals. This hybridization enhances the bonding interactions between TM and Al atoms, thereby effectively improving the structural stability and mechanical properties of the alloys. In summary, Al_5_TM alloys exhibit a combination of structural stability, excellent mechanical properties, and good electrical conductivity, demonstrating great potential for applications as high-temperature structural materials or advanced functional materials.

## Figures and Tables

**Table 1:** Calculated lattice parameters (Å), density *ρ* (g/cm^3^), and formation energy *E_f_* (eV/atom) of Al_5_TM alloys.

Space Groups	Al_5_TM	*a*	*c*	*ρ*	*E_f_*	Ref.
*R*32	Al_5_Mo	4.95	13.16	4.125	−0.295	this work
*P*6_3_	Al_5_Mo	4.92	8.89		−0.231	[26]
*R*32	Al_5_Nb	5.05	13.35	3.851	−0.241	this work
*R*32	Al_5_Os	4.73	13.90	6.015	−0.186	this work
*R*32	Al_5_Re	4.84	13.29	5.923	−0.176	this work
*R*32	Al_5_Ru	4.76	13.73	4.364	−0.245	this work
*R*32	Al_5_Ta	5.04	13.35	5.353	−0.183	this work
*R*32	Al_5_Tc	4.87	13.17	4.268	−0.257	this work
*R*32	Al_5_Ti	5.02	13.45	3.096	−0.206	this work
*P*6_3_	Al_5_W	4.92	8.89		−0.151	[26]

**Table 2:** Calculated elastic constants *C_ij_* (GPa) of Al_5_TM alloys.

Al_5_TM	*C*_11_	*C*_12_	*C*_13_	*C*_14_	*C*_22_	*C*_23_	*C*_33_	*C*_44_	*C*_55_	*C*_66_
Al_5_Mo	216	58	64	−3	216	64	250	93	93	79
Al_5_Nb	199	48	55	−8	199	55	255	94	94	76
Al_5_Os	250	78	87	11	250	87	179	29	29	86
Al_5_Re	225	68	101	13	225	101	225	61	61	79
Al_5_Ru	215	75	76	12	215	76	181	26	26	70
Al_5_Ta	202	49	58	−9	202	58	263	95	95	77
Al_5_Tc	206	66	69	13	206	69	216	63	63	68
Al_5_Ti	124	66	43	−10	124	43	224	67	67	29

**Table 3:** Calculated shear modulus *G* (in GPa), bulk modulus *B* (in GPa), Young’s modulus *E* (in GPa), *B*/*G*, and Poisson’s ratio *v* of Al_5_TM alloys.

Al_5_TM	*G*	*B*	*E*	*B*/*G*	*v*
Al_5_Mo	85.5	116.3	205.9	1.36	0.21
Al_5_Nb	84.9	106.3	201.1	1.25	0.18
Al_5_Os	50.1	130.3	133.3	2.60	0.33
Al_5_Re	65.8	134.6	169.8	2.04	0.29
Al_5_Ru	44.2	118.3	117.9	2.68	0.33
Al_5_Ta	85.7	109.9	204.1	1.28	0.19
Al_5_Tc	65.7	113.7	165.2	1.73	0.26
Al_5_Ti	48.2	84.9	121.7	1.76	0.26

**Table 4:** Calculated longitudinal elastic wave velocity $v_{l}$ (in m/s), transverse elastic wave velocity $v_{t}$ (in m/s), and average sound velocity $v_{m}$ (in m/s) of Al_5_TM alloys.

Al_5_TM	$v_{l}$	$v_{t}$	$v_{m}$
Al_5_Mo	7542	4590	5070
Al_5_Nb	7549	4696	5174
Al_5_Os	5724	2886	3236
Al_5_Re	6127	3334	3719
Al_5_Ru	6373	3183	3571
Al_5_Ta	6454	3994	4405
Al_5_Tc	6866	3921	4359
Al_5_Ti	6942	3947	4388

## Data Availability

The raw data supporting the conclusions of this article will be made available by the authors upon request.
